# Ustekinumab Dosing Individualization in Crohn’s Disease Guided by a Population Pharmacokinetic–Pharmacodynamic Model

**DOI:** 10.3390/pharmaceutics13101587

**Published:** 2021-09-30

**Authors:** Jurij Aguiar Zdovc, Jurij Hanžel, Tina Kurent, Nejc Sever, Matic Koželj, Nataša Smrekar, Gregor Novak, Borut Štabuc, Erwin Dreesen, Debby Thomas, Tomaž Vovk, Barbara Ostanek, David Drobne, Iztok Grabnar

**Affiliations:** 1 Department of Biopharmaceutics and Pharmacokinetics, Faculty of Pharmacy, University of Ljubljana, 1000 Ljubljana, Slovenia; jurij.aguiar.zdovc@ffa.uni-lj.si (J.A.Z.); tomaz.vovk@ffa.uni-lj.si (T.V.); 2 Department of Gastroenterology, University Medical Centre Ljubljana, 1000 Ljubljana, Slovenia; jurij.hanzel@gmail.com (J.H.); tina.kurent@gmail.com (T.K.); nejc.sever@gmail.com (N.S.); kozelj.matic@gmail.com (M.K.); nsmreki@gmail.com (N.S.); grega84@gmail.com (G.N.); borut.stabuc@gmail.com (B.Š.); david.drobne@gmail.com (D.D.); 3 Department of Internal Medicine, Medical Faculty, University of Ljubljana, 1000 Ljubljana, Slovenia; 4 Department of Pharmaceutical and Pharmacological Sciences, KU Leuven, 3000 Leuven, Belgium; erwin.dreesen@kuleuven.be (E.D.); debby.thomas@kuleuven.be (D.T.); 5 Department of Pharmacy, Uppsala University, 751 23 Uppsala, Sweden; 6 Department of Clinical Biochemistry, Faculty of Pharmacy, University of Ljubljana, 1000 Ljubljana, Slovenia; barbara.ostanek@ffa.uni-lj.si

**Keywords:** ustekinumab, inflammatory bowel disease, fecal calprotectin, pharmacokinetics-pharmacodynamics, therapeutic drug monitoring

## Abstract

Ustekinumab is a monoclonal antibody used in Crohn’s disease (CD). Dose optimization in case of non-response and the role of pharmacokinetic–pharmacodynamic (PK-PD) monitoring remain unresolved dilemmas in clinical practice. We aimed to develop a population PK-PD model for ustekinumab in CD and simulate efficacy of alternative dosing regimens. We included 57 patients and recorded their characteristics during 32 weeks after starting with ustekinumab therapy. Serum ustekinumab concentration was prospectively measured and fecal calprotectin (FC) concentration was used to monitor the disease activity. Ustekinumab PK-PD was described by a two-compartment target-mediated drug disposition model linked to an indirect response model. Lower fat-free mass, higher serum albumin, previous non-exposure to biologics, *FCGR3A*-158 V/V variant and lower C-reactive protein were associated with higher ustekinumab exposure. Model-based simulation suggested that 41.9% of patients receiving standard dosing achieve biochemical remission at week 32. In patients not achieving remission with standard dosing at week 16, transition to 4-weekly subcutaneous maintenance dosing with or without intravenous reinduction resulted in comparably higher remission rates at week 32 (51.1% vs. 49.2%, respectively). Our findings could be used to guide stratified ustekinumab treatment in CD, particularly in patients with unfavorable characteristics, who might benefit from early transition to 4-weekly maintenance dosing.

## 1. Introduction

Crohn’s disease (CD) is a debilitating, relapsing-remitting, incurable inflammatory disease of the digestive tract [1]. Treatment goals have shifted from symptomatic improvement to a combination of endoscopic and clinical remission [2]. Biomarker remission was identified as an adjunct treatment goal with fecal calprotectin (FC) being sensitive for endoscopic disease activity [3,4].

Ustekinumab is an IgG1κ monoclonal antibody that binds with high affinity to the p40 subunit shared by interleukin 12 (IL12) and interleukin 23 (IL23) [5]. Standard dosing with weight-based intravenous induction followed by 90 mg subcutaneous injections every 8 weeks is not effective for all patients, with real-world studies reporting that only up to one half of the patients achieve clinical remission, and up to 25% of patients achieve endoscopic remission [6,7,8,9,10,11]. The exposure–response relationship between peak ustekinumab serum concentration after the induction dose and endoscopic remission at 24 weeks suggests that a subset of patients not responding to conventional dosing may benefit from dose escalation [11]. Recent real-world studies assessing off-label intensified maintenance therapy with 90 mg administered every four weeks in patients with insufficient response have shown favorable rates of clinical, biochemical and endoscopic remission [12,13,14,15].

It remains unclear which patients are best suited for dose optimization, what is the most suitable dosing strategy (intravenous re-induction or dosing interval shortening for subcutaneous therapy) and how pharmacokinetic–pharmacodynamic (PK-PD) monitoring could improve the effectiveness of ustekinumab. Moreover, clinical PK studies of ustekinumab in CD are scarce and data on how demographic, pathophysiological, and genetic factors impact the drug’s PK and PD, are limited.

Our objective was to develop a population PK-PD model linking ustekinumab dosing regimen to the time course of FC concentrations. Through model-based simulations, which integrate both PK and PD data, we aimed to explore different dosing strategies, which are increasingly used in daily clinical practice.

## 2. Materials and Methods

### 2.1. Patients and Study Design

This was a prospective observational study in patients with CD starting treatment with ustekinumab at a single tertiary referral center. The study design and clinical results have partially been reported elsewhere [11]. Briefly, consecutive patients aged ≥18 years with CD who started treatment with ustekinumab between October 2017 and June 2019 were screened for eligibility and included in the current study, regardless of baseline endoscopic disease activity. They received a weight-based intravenous induction dose (≤55 kg: 260 mg; 55–85 kg: 390 mg; >85 kg: 520 mg) infused over one hour at baseline, followed by a fixed subcutaneous maintenance dose of 90 mg every 8 weeks. The duration of follow-up was 32 weeks.

### 2.2. Pharmacokinetic and Pharmacodynamic Data

Serum samples for the PK analysis were prospectively collected at baseline, 1 h after the end of the intravenous infusion (peak), and subsequently at weeks 2, 4, 8, 9, 10, 12, 16, 20, 24, and 32. Fecal samples of the first morning bowel movement were collected for the PD analysis at baseline, and subsequently at weeks 8, 16, 24, and 32. Ileocolonoscopies were performed within 2 months before starting ustekinumab to determine the presence of mucosal ulcerations to define endoscopically active luminal disease at baseline. Blood samples for genotyping analyses were collected at baseline.

Serum unbound ustekinumab concentration (hereafter referred to as ustekinumab concentration) was measured with a validated enzyme-linked immunosorbent assay (ELISA, ImmunoGuide^®^, AybayTech Biotechnology, Ankara, Turkey), with a lower limit of quantification (LOQ) at 0.35 μg/mL [11]. Antibodies to ustekinumab were measured with a drug-tolerant ELISA in serum samples with ustekinumab concentration <1 μg/mL [10]. FC was measured with a Calprest ELISA assay (Eurospital, Triest, Italy) with a measurement range of 15.6–500 mg/kg.

Genomic DNA was isolated from whole blood collected in EDTA tubes by using a FlexiGene DNA kit (Qiagen, Hilden, Germany). The concentration and purity of DNA were measured on NanoDrop™ One/OneC Microvolume UV-Vis Spectrophotometer (Thermo Fischer Scientific, Waltham, MA, USA). Genotyping of single nucleotide polymorphisms (SNPs) was performed by TaqMan^®^ Pre-Designed SNP Genotyping Assays (Thermo Fischer Scientific, Waltham, MA, USA) on a LightCycler 480II real-time polymerase chain reaction instrument (Roche, Basel, Switzerland) according to manufacturer’s recommendations. SNPs were selected based on previously identified associations with ustekinumab treatment outcomes in psoriasis or a possible role in the mediation of IgG clearance [16,17,18]. The analyzed SNPs and the specific assays used were: *IL12B* (rs3212227, assay ID: C_2084293_10; rs3213094, assay ID: C_29927086_10; and rs6887695, assay ID: C_1994992_10), *FCGR2A* (rs1801274, assay ID: C_9077561_20) and *FCGR3A* (rs396991, assay ID: C_25815666_10). To validate our results, 20% of the samples were re-genotyped for each SNP, and the results were found to be reproducible with no discrepancies noted.

Demographic characteristics (age, weight, height, sex), clinical data (co-morbidities, disease history and disease location, concomitant and previous treatment) and biochemical markers (C-reactive protein (CRP), serum albumin) were recorded. Fat-free mass (FFM) was determined using the Janmahasatian model [19].

### 2.3. Statistical Analysis and Pharmacokinetic–Pharmacodynamic Modeling

Descriptive statistics were used to present the data as non-normally distributed with medians and inter-quartile ranges (IQR). The nonlinear mixed-effects methodology and NONMEM^®^ software (version 7.3, Icon Development Solutions, Ellicott City, MD, USA) was used to analyze the PK and PD data and develop a population PK-PD model, linking ustekinumab concentrations, patients’ characteristics and FC at all time points.

A population PK model was developed using the ustekinumab concentrations converted to nanomolar concentration, assuming its molecular weight of 149 kDa. A linear one- and two-compartment models, and approximations of the target mediated drug disposition (TMDD) model were tested [20,21,22,23]. First-order absorption of ustekinumab was assumed after the subcutaneous dosing. Considering the concentration of ustekinumab target (p40 subunit of IL12 and IL23) was not measured, it was modeled as a latent variable in TMDD models. The PK studies in monkeys report a bi-exponential decline of the cytokines containing the p40 subunit (IL12 and IL23) [24,25]. Therefore, the PK data were extracted from these studies and PK parameters for the IL12 and IL23 were estimated and allometrically scaled to human. These estimates were used as initial estimates of the PK parameters of the target disposition in the TMDD models. One- and two-compartment models were tested to describe the distribution of the target. 

Additive, proportional and combination (additive + proportional) error models were tested for residual variability. The logit transformation was used to describe the interindividual variability (IIV) of fraction of absorbed ustekinumab and the exponential model was used to describe the IIV of other parameters. The model with the lowest Akaike information criterion (AIC) was used as a base PK model (Table S1), for the subsequent covariate model building.

To explain the estimated IIV in the PK parameters, the candidate parameter–covariate relationships were selected based on scientific plausibility, previously reported relationships, and trends in correlation plots between individual PK parameters and covariates. Stepwise covariate procedure (*p* < 0.05 in the forward inclusion, *p* < 0.01 in the backward elimination) was used to test the significance of parameter–covariate relationships. To estimate the effect of the body size, FFM was chosen over total body weight, considering the distribution of monoclonal antibodies is predominantly limited to extracellular fluids. The evaluated continuous covariates were: disease duration, FFM, baseline serum CRP concentration and serum albumin concentration. The evaluated categorical covariates were: previous biological therapy (bio-naïve), smoking, and SNPs in *IL12B* (rs3212227, rs3213094, rs6887695), *FCGR2A* (rs1801274) and *FCGR3A* (rs396991). Linear and power model were tested for continuous covariates and dominant and recessive grouping combinations were tested for SNPs.

The individual PK parameters obtained from the final PK model were used for the subsequent PD analysis [26], to describe the relationship between ustekinumab PK, target disposition and FC concentration. The bio-phase distribution model, indirect response model and signal transduction (transit compartment indirect response) model were tested to describe the delay between ustekinumab PK and PD [27]. Exponential model, as well as Box-Cox transformation were tested to describe the IIV of baseline FC concentration (FC_0_).

Laplacian estimation with interaction was used for parameter estimation. The M3 method was used for data below the lower or above the upper LOQ [28]. AIC was used for model comparison. The model was internally validated with a visual predictive check (VPC, n = 2000) and parameter uncertainty was assessed with bootstrap with replacement method (n = 2000).

### 2.4. Model Based Simulations

The final PK-PD model and NONMEM^®^ were used for simulation of various treatment regimens and scenarios of PK and PD monitoring. The proportion of patients achieving biochemical remission (FC < 100 mg/kg) at week 8, week 16, week 24 and week 32 after the first dose was estimated for every treatment regimen, to compare the efficacy. This cut-off was chosen based on test characteristics identified by meta-analyses [29], with emphasis on studies using the same assay as our center, considering the large inter-assay variability [30].

First, a virtual population of 10,000 CD patients was created, which resembled the observed cohort regarding the distributions and correlations of patients’ characteristics. Subsequently, several clinically relevant scenarios were simulated, as follows:

(a). All patients received standard ustekinumab treatment, with weight-based induction dose at baseline, followed by fixed 90 mg maintenance doses every eight weeks (standard treatment);
(b). All patients received weight-based induction dose at baseline followed by fixed 90 mg maintenance doses every four weeks;
(c). All patients received weight-based induction doses every eight weeks;
(d). Patients receiving standard treatment who were not in remission at week 16, switched to maintenance doses every four weeks from week 20;
(e). Patients receiving standard treatment who were not in remission at week 16 received a weight-based reinduction dose at week 16, and continued with maintenance doses every eight weeks;
(f). Patients receiving standard treatment who were not in remission at week 16 received a weight-based reinduction dose at week 16 and switched to maintenance doses every four weeks from week 20.

R software, version 4.0.2 (R Development Core Team, Vienna, Austria), RStudio version 1.3.1073 (RStudio Team, PBC, Boston, MA, USA) and packages mvtnorm, plyr, dplyr, reshape2 and ggplot2 were used for creating the virtual patient population, data wrangling and visualizations.

## 3. Results

### 3.1. Baseline Patient Characteristics

The study included 57 patients: the median disease duration was 14 years (IQR 7–22), 66.7% (38/57) had been previously exposed to biological therapy, and 77.2% (44/57) had endoscopically active disease at baseline (Table 1). All patients completed the study.

### 3.2. Pharmacokinetic and Pharmacodynamic Data

A total of 574 serum samples was available for ustekinumab measurement. Five samples had an ustekinumab concentration below the lower LOQ, and none of the analyzed samples had measurable antibodies to ustekinumab. SNPs were analyzed for all patients, and all genotype frequencies were in Hardy–Weinberg equilibrium (Table 1). A total of 224 samples was available for FC measurement. There were 15 samples with FC concentration below the lower LOQ and 11 samples above the upper LOQ.

### 3.3. Pharmacokinetic–Pharmacodynamic Model

**Figure 1:**
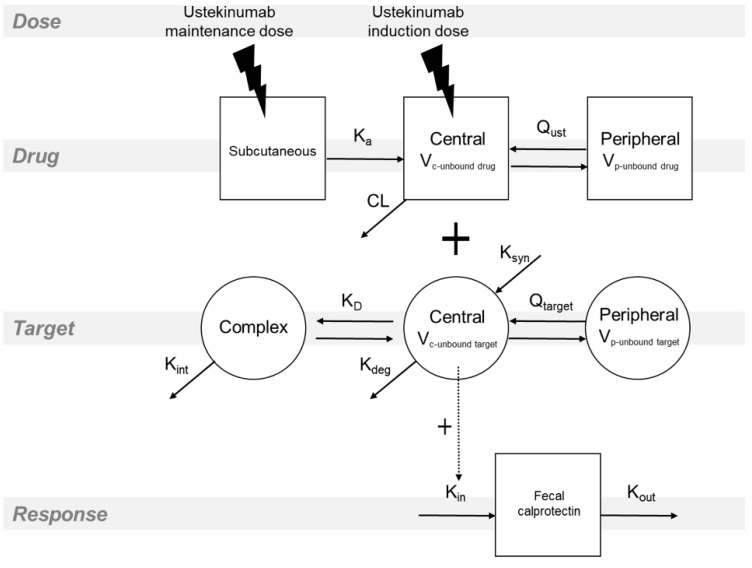
Schematic of the population pharmacokinetic–pharmacodynamic model. A first-order absorption of ustekinumab (drug) is assumed after the subcutaneous administration. The distribution of the unbound drug and unbound target is described with the two-compartment models. The binding is assumed in the central compartment and is described with the quasi-equilibrium approximation of the target-mediated drug disposition model. The stimulating effect of the unbound target on the fecal calprotectin is described with the indirect response model. Ka: ustekinumab absorption rate constant after the subcutaneous administration; V_c_: volume of distribution in the central compartment; V_p_: volume of distribution in the peripheral compartment; Q_UST_: intercompartmental clearance of unbound ustekinumab; CL: clearance of unbound ustekinumab; K_syn_: rate constant of target synthesis; K_deg_: rate constant of target degradation; K_D_: constant of the equilibrium between unbound drug, unbound target and drug-target complex; K_int_: rate constant of internalization of the drug-target complex into the cells; Q_target_: intercompartmental clearance of unbound target; K_in_: rate constant of calprotectin production; K_out_: rate constant of fecal calprotectin degradation.

A quasi-equilibrium approximation of the TMDD model [23] best described the time profile of the ustekinumab concentration (Table S1). The model (Figure 1) assumes rapid binding of ustekinumab to the target antigen and was extended with distribution of the unbound ustekinumab and unbound target into a peripheral compartment since it resulted in an improved fit (Table S1). The binding was assumed in the central compartment. Estimated parameters comprised linear ustekinumab PK, linear target PK and parameters related to the binding of ustekinumab to the target (Table 2). The terminal half-life of ustekinumab after the induction dose in a typical patient was estimated at 17 days and the volume of distribution was low, with volumes of central and peripheral compartment of ustekinumab estimated at 3.57 L and 3.30 L, respectively. There was a high IIV of the rate constant of target synthesis (K_syn_), with the coefficient of variation estimated at 99.5%. 

Covariates associated with PK parameters were as follows: ustekinumab clearance was higher with increasing FFM, decreasing serum albumin, and in patients with previous exposure to biologics (Table 2). A power model described the increase of volumes of the central and peripheral compartment of ustekinumab with increasing FFM (Table 2). The fraction of absorbed ustekinumab was higher in valine homozygous (V/V) patients of rs396991 *FCGR3A* polymorphism, compared to phenylalanine homozygotes (F/F) and heterozygotes (V/F) combined (Table 2, Figure S1). Additionally, K_syn_ increased with increasing baseline CRP (Table 2).

**Figure 2:**
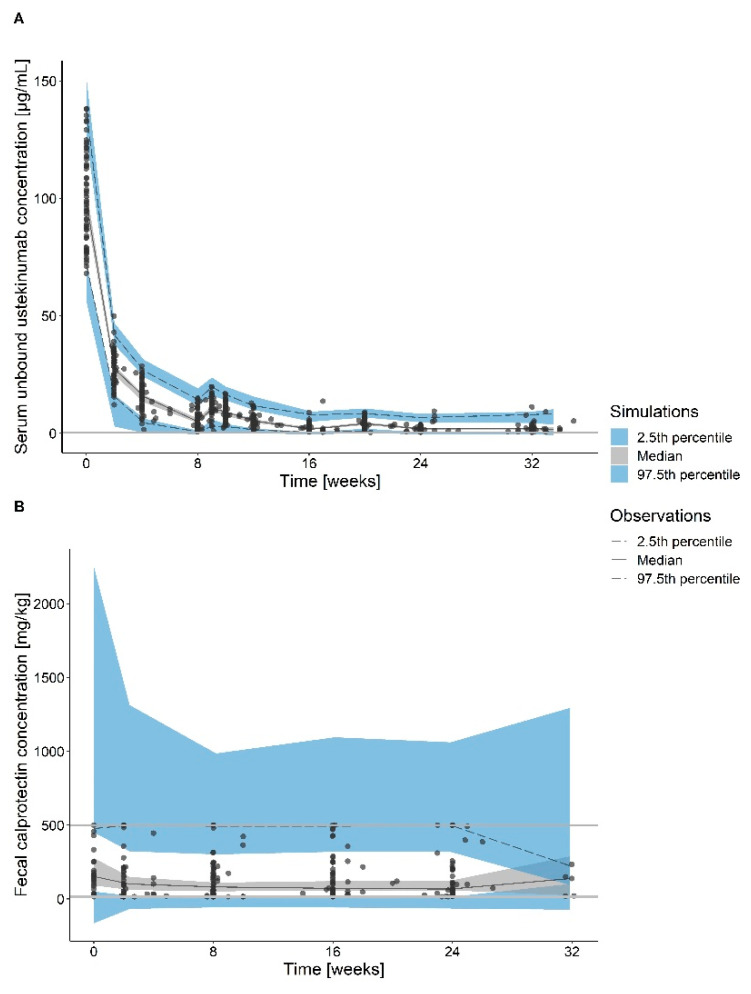
Visual predictive check (n = 2000) of the final pharmacokinetic (**A**) and final pharmacodynamic (**B**) models. Observations (points), medians (black lines), 5th and 95th percentiles (dashed black lines), 95% confidence intervals of the simulated medians (grey shaded areas), 2.5th and 97.5th percentiles (blue shaded areas).

The concentration of the unbound target was linked to the FC concentration via the indirect response model, which best described the stimulating effect of the unbound target on the FC production (Table 3). Thus, the model explains the ustekinumab effect on the decrease in FC concentration via the intermediate target layer (Figure 1). The initial concentration of the target and FC were modelled as K_syn_/K_deg_ and FC_0_, respectively. In the absence of ustekinumab, steady concentration of the target and FC is assumed. In contrast, when ustekinumab is administered and bound to the target, the unbound target concentration decreases, which inhibits the FC production. Exponential model best described the IIV on FC_0_ and patients who started ustekinumab due to endoscopically active disease, had higher baseline FC concentration, which was accounted for in the PD model as a binary covariate on FC_0_ (Table 3). There was a good agreement between model predictions and the observed data (Figure 2 and Figure S2).

### 3.4. Simulations

The simulation of patients receiving standard ustekinumab regimen demonstrates that patients with weight ≤55 kg have lower ustekinumab concentration in the induction phase, and higher ustekinumab concentration in the maintenance phase, compared to patients with weight >55 kg. In addition, patients in the lowest weight group had higher unbound p40 and FC concentrations during the induction phase (Figure S3). 

After the first ustekinumab administration, the unbound target rapidly decreases. A subsequent increase in the target concentration is observed, due to the constant target synthesis. A faster increase in the target concentration in patients with weight >55 kg is observed, compared to patients with weight ≤55 kg, because of the fixed 90 mg subcutaneous dose in the maintenance phase and thus higher exposure to ustekinumab of patients with weight ≤55 kg (Figure S3). In addition, initial target concentration influences the peak ustekinumab concentration. A patient with the 10-fold higher K_syn_ compared to the typical value, would have approximately 15 μg/mL lower peak ustekinumab concentration (Figure S4).

The dynamics of the FC are similar across the weight groups, with faster increase of the FC concentration in higher weight groups (Figure S3). Patients with the endoscopically active disease at baseline had higher FC concentration, and only these patients were considered for the rest of simulations. 

**Figure 3:**
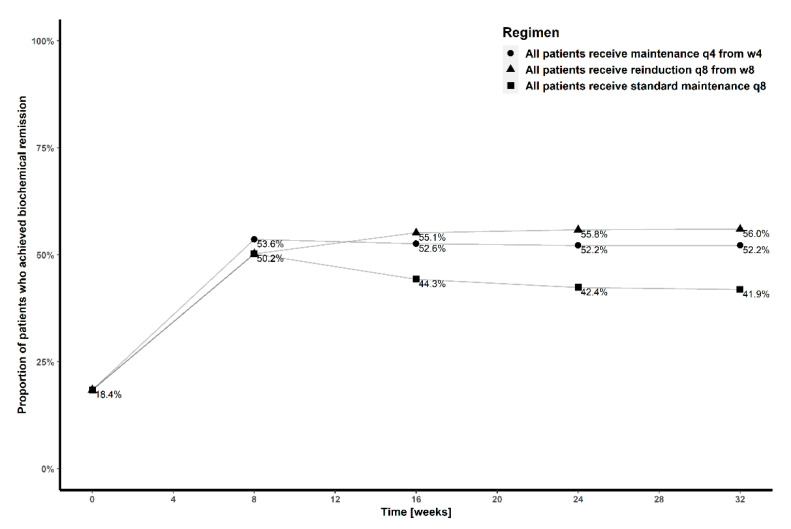
Proportion of patients achieving biochemical remission at weeks 8, 16, 24 and 32, with standard treatment (■), treatment with initial induction, followed by maintenance dosing every four weeks from week 4 (●) and induction dosing every eight weeks (▲).

**Figure 4:**
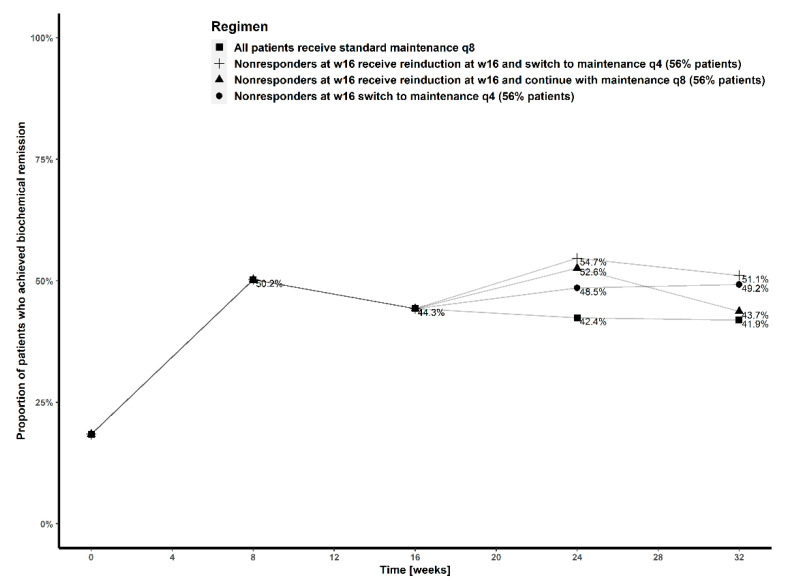
Proportion of patients who achieved biochemical remission at weeks 8, 16, 24 and 32, with standard treatment (■), treatment where patients not in remission at week 16 received maintenance dosing every four weeks from week 20 (●), treatment where patients not in remission at week 16 received intravenous reinduction at week 16 and continued with maintenance dosing every eight weeks (▲), and treatment where patients not in remission at week 16 received intravenous reinduction at week 16, followed by maintenance dosing every four weeks from week 20 (+).

The proportions of patients in remission at week 32 receiving standard treatment with maintenance dosing every 8 weeks, treatment with initial induction and subsequent maintenance dosing every four weeks from week 4 or treatment with induction dosing every eight weeks are 41.9%, 52.2% and 56.0%, respectively (Figure 3). In patients not achieving remission with standard treatment at week 16, transition to subcutaneous maintenance dosing every 4 weeks with or without intravenous reinduction resulted in similar biochemical remission at week 32 (51.1% vs. 49.2%, respectively), while reinduction and continuation with maintenance dosing every 8 weeks was comparable to standard dosing (Figure 4).

## 4. Discussion

Clinical PK studies of ustekinumab are relatively scarce, and there is currently no consensus on the optimal management of inadequate response to treatment. To our knowledge, this is the first study to present a semi-mechanistic PK-PD model linking ustekinumab PK, CD patients’ clinical characteristics, binding to p40 subunit and FC dynamics. Additionally, we are the first to evaluate the influence of genetic factors on ustekinumab PK-PD in CD. Model-based simulations suggest the possibility of achieving better outcomes with intensified dosing and provide further insight into managing patients with insufficient response at week 16.

Building on previous work identifying an exposure–response relationship for ustekinumab for biochemical and endoscopic outcomes [8,10,11,31], our PK-PD model suggests that rates of biochemical remission could be improved by using intensified dosing regimens to increase ustekinumab exposure (Figure 4). A subgroup of patients at risk of insufficient exposure to ustekinumab (albumin < 43 g/L, previously exposed to biological treatment, V/F or F/F genotype of rs396991, CRP > 5 mg/L) were predicted to benefit the most from dosing escalation. These patients could also be considered for potential proactive dosing escalation in future studies (Figures S5 and S6). The proportion of patients achieving biochemical remission decreased with an increasing number of unfavorable covariates, and this trend was attenuated by dosing escalation (Figure S6). 

Different approaches have been used in patients not responding or losing response to standard dosing of ustekinumab: dosing interval shortening [12,13,14,15], intravenous reinduction [32] or a combination of both [32,33]. These modifications were made at week 16 of treatment or later. Clinical remission, defined as a Harvey–Bradshaw Index ≤ 4, was reported in 28–40% of patients after interval shortening to every 4 weeks [12,13,14], CRP normalization occurred in 22% of patients [12]. The interpretation of endoscopic remission rates around 35% (4/11, 14/39) is confounded by low numbers and potential selection bias where patients with biochemically active disease did not undergo endoscopic assessment [12,14]. None of the studies conducted so far were designed and powered to compare the efficacy of different strategies. Simulations based on our model showed that switching to maintenance dosing every 4 weeks from week 16 leads to similar rates of biochemical remission at week 32 regardless of an additional intravenous reinduction dose. A single additional reinduction dose followed by maintenance every 8 weeks is expected to provide only transient benefit (Figure 4). This is broadly concordant with the findings of a retrospective real-world series, where 15/18 patients responded clinically to an additional intravenous infusion and 10/15 required escalated subcutaneous dosing in ongoing maintenance [32]. Even patients already on maximized subcutaneous dosing may benefit from an additional intravenous infusion, although none of these studies included PK assessment [33].

Our findings should be compared with the recently reported results of the STARDUST trial (NCT03107793) evaluating the efficacy of a treat-to-target (T2T) approach to guide treatment escalation decisions compared to standard of care (SOC) [34]. Clinical responders at week 16 underwent randomization, dosing in the T2T arm was first determined based on the change in endoscopic score from baseline and subsequently on combined clinical and biomarker (CRP, FC) monitoring. At week 48, endoscopic response (T2T: 37.7%; SOC: 29.9%) and remission (T2T: 11.4%; SOC: 14.5%) were not significantly different between the two groups. Notably, only 17% of patients in the T2T arm were on four-weekly dosing at week 48, although this percentage is also affected by the 20% discontinuation rate in this arm as patients not achieving treatment targets within four weeks of starting four-weekly dosing discontinued the study. It also remains to be seen what proportion of discontinuations was driven by clinical disease activity despite biochemical remission, as both were required to meet the target. Despite these results, dose escalation may still hold added benefit, particularly if applied early after induction to a selected subpopulation of patients unlikely to respond to standard dosing due to low drug exposure.

The estimated terminal half-life in our study is in accordance with PK studies in psoriasis, psoriatic arthritis, and IBD [35,36,37,38], reporting half-life between 19 and 22 days. The distribution of ustekinumab into tissues was limited, which is consistent with its high molecular weight and hydrophilic properties. Ustekinumab linear clearance was in range of reported clearance values in other studies in IBD [37,38,39], and the TMDD PK model is consistent with the recent findings of the TNF-mediated PK of infliximab in ulcerative colitis [40]. Notably, the elimination of ustekinumab in our model was captured in two catabolic pathways, distinct for IgG antibodies [41]: a linear, non-specific clearance, and a nonlinear, specific clearance, mediated by the binding of the antibody to its target. Therefore, a higher concentration of the target reflecting increased inflammatory burden may lead to accelerated ustekinumab clearance and lower ustekinumab concentration. In CD, the concentration of the p40 subunit is related to the disease activity, although the reported serum concentrations of IL12 and IL23 are highly variable [42,43].

The PK model additionally includes a bi-exponential decline of the concentration of the target, which reflects the possible distribution of the target to other tissues and is in line with the in vivo studies of IL12 and IL23 [24,25]. Thus, the initial binding of ustekinumab to the target in the serum may explain the influence of the target on the peak ustekinumab concentration and volume of distribution (Figure S4). The subsequent redistribution of the target from other tissues into the serum, as well as the constant target synthesis, may explain the target-mediated ustekinumab clearance. 

We have identified similar factors influencing ustekinumab PK compared to other studies [35,36,37,38]. Ustekinumab exposure was found to increase with decreasing FFM. However, dosing regimens seemed similarly efficient in patients stratified by the weight according to the induction dose (Figure S6). This indicates that the weight is not related with ustekinumab efficacy and is in line with the recent post hoc analysis of IM-UNITI study, assessing the impact of body mass index on clinical efficacy [44]. The exposure increased with increasing serum albumin, as well. A more severe disease may result in a leakage of the albumin and drug into the bowel. The correlation may additionally be explained by the role of FcRn in homeostatic regulation of both, IgG and albumin [45]. Moreover, ustekinumab exposure was higher in bio-naïve patients. Presumably, these patients are less likely to have a more severe type of disease compared to those who have previously failed therapy with other biologics. Therefore, bio-naïve patients may express lower target concentrations, which leads to lower ustekinumab clearance and higher exposure. 

We also observed higher fraction of absorbed ustekinumab, and higher exposure related with V/V variant of the rs396991 polymorphism, otherwise known as *FCGR3A*-158 V/F (Figure S1, Table 2). This is a functionally significant polymorphism in *FCGR3A* gene, which encodes the FcγRIIIa receptor. Our finding is highly interesting, since it mirrors the studies reporting association between the V/V genotype and better outcomes of CD treatment with infliximab [46,47]. The V variant is presumably related with higher affinity between FcγRIIIa and the IgG antibodies, compared to the F variant, which may result in altered binding at the absorption site and lead to a variable response to treatment in IBD [48]. However, further studies are necessary to clarify the clinical relevance and the mechanistic involvement of *FCGR3A*-158 polymorphism in ustekinumab PK-PD. 

The indirect response PD model for FC has been previously used in a PK-PD studies of infliximab and ustekinumab in CD [38,49]. Notably, the rates of FC degradation are similar, which may additionally account for the different FC measurement assays. In our study, all patients regardless of the endoscopic disease activity were included, and the presence of mucosal ulcerations at baseline was associated with higher baseline FC and CRP, indicating a higher inflammatory burden in patients with confirmed active luminal disease. Antibodies to ustekinumab were not detected in any of the tested samples, which is consistent with the low observed rates of immunogenicity in registration studies [50,51,52].

Specifically, our PK-PD model is in line with the recent study by Wang et al. [38], who reported a PK-PD model linking ustekinumab, FC and endoscopic outcomes, based on the sparse data from an exposure-response study by Verstockt et al. [10]. Notably, the estimated ustekinumab linear CL is similar (Wang et al.: 0.235 L/day; our study: 0.277 L/day), both models assumed the disposition of ustekinumab into the peripheral compartment and included similar covariates affecting the PK (Wang et al.: serum albumin, body weight; vs. our study: serum albumin, FFM, bio-naïve, *FCGR3A*-158, CRP). The difference in the percent of bio-naïve patients (Wang et al.: 5%; vs. our study: 33%) and the testing of the genetic data in our study may in part explain the different covariate model structure. The simulated median trough steady state ustekinumab concentration for standard regimen (Wang et al.: 1.3 μg/mL; vs. our study: 1.5 μg/mL) and regimen with maintenance dosing every 4 weeks (Wang et al.: 5.3 μg/mL vs. our study: 5.1 μg/mL) were similar, as well. 

Additionally, both studies used indirect response PD models with comparable K_out_ estimate for FC (Wang et al.: 0.0416 day^−1^; vs. our study: 0.0581 day^−1^), although there was a large observed difference in the average FC concentration due to the different assay [53], and in our model the FC concentration was linked to the latent target instead of the ustekinumab. The residual unexplained PD variability was relatively high in both studies, at more than 55%. Nevertheless, both studies suggested the potential benefit of the shorter interval of the maintenance dosing.

Our findings should be interpreted in the context of the study’s limitations. The model was developed in a small cohort of patients and the target concentration was not measured. Despite the plausibility of covariates explaining the PK of ustekinumab, which overlap with findings from other immune-mediated inflammatory diseases and models for other monoclonal antibodies in IBD, the model should be further validated in an independent cohort. Notwithstanding the correlation between endoscopic outcomes and FC concentrations, normalization of the latter is neither an independent treatment goal nor does its normalization guarantee endoscopic remission as observed in the STARDUST trial. Finally, outcomes of simulations based on our model should be regarded as hypothesis-generating for future study design and not used to inform clinical practice here and now.

In conclusion, this article presents a semi-mechanistic population PK-PD model for ustekinumab in CD, and the findings could be used to guide future attempts to personalize treatment with ustekinumab in CD. Especially patients with low serum albumin, previous failure of biological therapy, *FCGR3A*-158 V/F or F/F variant and high CRP might benefit from early optimization of ustekinumab therapy to 4-weekly maintenance dosing.

## Figures and Tables

**Table 1:** Patients’ characteristics at baseline (n = 57).

Characteristic	Value
Women, n (%)	32 (56)
Age at UST initiation, years, median (IQR)	49 (32–56)
Weight, kg, median (IQR)	70 (59–84)
Fat-free mass ^a^, median (IQR)	45 (39–62)
Height, cm, median (IQR)	169 (163–179)
Intravenous ustekinumab dose, n (%)	
260 mg	9 (15.8)
390 mg	35 (61.4)
520 mg	13 (22.8)
Disease duration, years, median (IQR)	14 (7–22)
Disease location, n (%)	
ileal (L1)	17 (29.8)
colonic (L2)	4 (7)
ileocolonic (L3)	36 (63.2)
upper gastrointestinal involvement (L4)	6 (10.5)
Fistulizing perianal disease, n (%)	10 (17.5)
History of CD-related surgery, n (%)	34 (59.6)
Smoking status, n (%)	
active smoking	10 (17.5)
previously smoking	12 (21.1)
never smoked	35 (61.4)
Previous biological therapy, n (%)	38 (66.7)
previous anti-TNF exposure	38 (66.7)
previous vedolizumab exposure	10 (17.5)
previous anti-TNF and vedolizumab exposure	9 (15.8)
Systemic steroids at baseline, n (%)	9 (15.8)
Topical steroids at baseline, n (%)	3 (5.3)
Immunomodulators at baseline, n (%)	5 (8.8)
azathioprine	4 (7)
methotrexate	1 (1.8)
Harvey–Bradshaw score, median (IQR)	6 (3–10)
Fecal calprotectin, mg/kg, median (IQR)	134 (53–213)
C-reactive protein, mg/L, median (IQR)	3 (3–11)
Serum albumin, g/L, median (IQR)	43 (41–44)
Endoscopically active disease at baseline, n (%)	44 (77.2)
Samples available (ustekinumab measurement), n	574
Samples available (Fecal calprotectin), n	224
Samples with fecal calprotectin below the limit of quantification, n (%)	15 (6.8)
Samples with fecal calprotectin above the limit of quantification, n (%)	11 (5)
Genotype frequencies	
*IL12B* rs3212227, n (%)	
A/A	37 (64.9)
A/C	17 (29.8)
C/C	3 (5.3)
*IL12B* rs3213094, n (%)	
C/C	37 (64.9)
C/T	17 (29.8)
T/T	3 (5.3)
*IL12B* rs6887695, n (%)	
G/G	22 (38.6)
C/G	25 (43.9)
C/C	10 (17.5)
*FcGR2A* rs1801274, n (%)	
A/A	21 (36.8)
A/G	29 (50.9)
G/G	7 (12.3)
*FcGR3A* rs396991, n (%)	
A/A	21 (36.8)
A/C	31 (54.4)
C/C	5 (8.8)

CD—Crohn’s disease; IQR—interquartile range; TNF—tumor necrosis factor; UST—ustekinumab. ^a^ The fat-free mass was predicted using the semi-mechanistic model developed by Janmahasatian et al. [19].

**Table 2:** Base and final pharmacokinetic model parameter estimates.

Parameter (Units)	Base Model	Final Model	
	Estimate	Estimate	Bootstrap Median (95% CI)
Ustekinumab pharmacokinetics			
K_a_ (day^−1^)	0.518	0.381	0.380 (0.341–0.422)
CL (L/day) ^a^	0.264	0.277	0.275 (0.259–0.294)
FFM on CL	/	0.598	0.596 (0.539–0.673)
bio-naïve on CL	/	−0.227	−0.232 (−0.280–−0.192)
Serum albumin on CL	/	−0.0165	−0.0170 (−0.0224–−0.0127)
V_c_ (L) ^b^	2.18	3.57	3.56 (3.41–3.70)
FFM on V_c_	/	0.590	0.587 (0.534–0.644)
Q (L/day)	20.1	1.89	1.88 (1.69–2.11)
V_p_ (L) ^c^	5.04	3.30	3.27 (3.01–3.52)
FFM on V_p_	/	0.586	0.581 (0.512–0.660)
Fraction absorbed, F (%)	71.7	/	/
*FCGR3A-158* V/V	/	88.8	88.8 (86.1–92.4)
*FCGR3A-158* V/F, F/F	/	71.0	70.8 (65.3–75.8)
Target pharmacokinetics			
K_syn_ (nmol/L × day^−1^) ^d^	1.65 × 10^−8^	9.86 × 10^−9^	9.85 × 10^−9^ (8.75 × 10^−9^–1.09 × 10^−8^)
Serum CRP on K_syn_	/	0.0846	0.0843 (0.0772–0.0883)
K_deg_ (day^−1^)	1.85 × 10^−10^	9.26 × 10^−10^	9.26 × 10^−10^ (8.53 × 10^−10^–1.06 × 10^−9^)
V_c–target_ (L)	18.8	2.44	2.44 (2.27–2.81)
Q_target_ (L/d)	0.752	0.493	0.488 (0.440–0.539)
V_p–target_ (L)	22.6	11.0	10.9 (9.87–12.0)
Binding			
K_int_ (day^−1^)	1.71 × 10^−7^	2.83 × 10^−6^	2.82 × 10^−6^ (2.56 × 10^−6^–3.15 × 10^−6^)
K_d_ (nmol/L)	0.350	0.168	0.168 (0.154–0.196)
Interindividual variability			
IIV CL (%, CV) ^e^	27.9 (13)	18.0 (16)	18.0 (16.0–20.1)
IIV V_c_ (%, CV) ^e^	32.9 (16)	9.79 (22)	9.79 (8.88–10.8)
IIV V_p_ (%, CV) ^e^	21.5 (23)	24.1 (24)	23.6 (19.7–26.0)
IIV F (%, SD) ^e^	16.6 (21)	17.3 (22)	17.4 (15.8–20.2)
IIV K_syn_ (%, CV) ^e^	105 (25)	99.2 (27)	98.4 (83.4–110)
Residual variability			
Additive RUV (nmol/L) ^e^	4.46 (18)	4.55 (17)	4.58 (4.09–5.86)
Proportional RUV (%) ^e^	7.84 (18)	7.77 (17)	7.74 (6.94–8.55)

CI—confidence interval; K_a_—ustekinumab absorption rate constant after subcutaneous administration; CL—clearance of ustekinumab; FFM—fat-free mass; V_c_—volume of distribution in central compartment; Q—intercompartmental clearance; V_p_—volume of distribution in peripheral compartment; F—fraction of absorbed ustekinumab after subcutaneous administration; K_syn_—rate constant of target synthesis; CRP—C-reactive protein; K_deg_—rate constant of target degradation; K_int_—elimination rate constant due to binding; K_d_—equilibrium constant; IIV—interindividual variability; CV—coefficient of variation; SD—standard deviation; RUV—residual unexplained variability. ^a^ $CL\left(\frac{L}{day}\right)=0.277\times {\left(\frac{FFM}{45}\right)}^{0.598}\times \left(1-0.0165\times \left(Serum\;albumin-43\right)\right)\times \left(1-0.227\times bio-naïve\right)$. ^b.^ $V_{c}\;\left(L\right)=3.57\times {\left(\frac{FFM}{45}\right)}^{0.590}$ ^c.^ $V_{p}\;\left(L\right)=3.30\times {\left(\frac{FFM}{45}\right)}^{0.586}$. ^d.^ $K_{syn}\;\left(\frac{nmol}{L}\times day^{-1}\right)=9.86\times 10^{-9}\times \left(1+0.0846\times \left(CRP-3\right)\right)$. ^e.^ For variability terms % shrinkage is presented in brackets.

**Table 3:** Final pharmacodynamic model ^a^ parameter estimates.

Parameter (Units)	Estimate	Bootstrap Median (95% CI)
K_out_ (day^−1^)	0.0581	0.0641 (0.0249–0.110)
FC_0_ (mg/kg)		
Patients without ulcers at baseline	102	105 (54.4–188)
Patients with ulcers at baseline	213	214 (157–287)
E_max_ (%)	219	227 (128–442)
C_50_ (nmol/L)	2.46	2.56 (0.413–13.9)
*Interindividual variability*		
IIV FC_0_ (%) ^b^	99.0 (2)	98.0 (75.0–128)
*Residual variability*		
Proportional RUV (%) ^b^	57.3 (17)	56.4 (48.8–65.0)

CI—confidence interval; K_out_—fecal calprotectin degradation rate constant; FC_0_—baseline fecal calprotectin concentration; E_max_—maximum effect; C_50_—target concentration at half-maximum effect; IIV—interindividual variability; RUV—residual unexplained variability. ^a.^ $\frac{dFC}{dt}=\frac{FC_{0}\times K_{out}}{1+\frac{E_{max}\times \frac{K_{syn}}{K_{deg}}}{C_{50}+\frac{K_{syn}}{K_{deg}}}}\times \left(1+\frac{E_{max}\times unbound\;target\;concentration}{C_{50}+unbound\;target\;concentration}\right)-K_{out}\times FC$. ^b.^ For variability terms % shrinkage is presented in brackets.

## Data Availability

The data presented in this study are available upon reasonable request from the corresponding author.

## Supplementary Materials

The following are available online at https://www.mdpi.com/article/10.3390/pharmaceutics13101587/s1, Figure S1: Fraction of absorbed ustekinumab after subcutaneous administration according to *FCGR3A* rs396991 polymorphism, Figure S2: Diagnostic plots of the final pharmacokinetic and pharmacodynamic model, Figure S3: A population simulation using the final pharmacokinetic–pharmacodynamic model and representative virtual patient population, Figure S4: Serum unbound ustekinumab concentration time-profile in a patient with high, typical, or low target synthesis rate constant, Figure S5: Simulated ustekinumab and fecal calprotectin concentration time-profile of a typical patient according to ustekinumab exposure and target synthesis, Figure S6: Proportion of patients who achieved biochemical remission at week 32 stratified by each covariate, Table S1: Comparison of the tested base pharmacokinetic models, Supplementary information S1: NONMEM control stream for the population PK and PD model.

## References

[1] Torres J., Mehandru S., Colombel J.-F., Peyrin-Biroulet L. (2017). Crohn’s disease. Lancet.

[2] Peyrin-Biroulet L., Sandborn W., Sands B.E., Reinisch W., Bemelman W., Bryant R.V., D’Haens G., Dotan I., Dubinsky M., Feagan B. (2015). Selecting Therapeutic Targets in Inflammatory Bowel Disease (STRIDE): Determining Therapeutic Goals for Treat-to-Target. Am. J. Gastroenterol..

[3] DʼHaens G., Ferrante M., Vermeire S., Baert F., Noman M., Moortgat L., Geens P., Iwens D., Aerden I., Van Assche G. (2012). Fecal calprotectin is a surrogate marker for endoscopic lesions in inflammatory bowel disease. Inflamm. Bowel Dis..

[4] Mosli M.H., Zou G., Garg S.K., Feagan S.G., MacDonald J.K., Chande N., Sandborn W.J., Feagan B.G. (2015). C-Reactive Protein, Fecal Calprotectin, and Stool Lactoferrin for Detection of Endoscopic Activity in Symptomatic Inflammatory Bowel Disease Patients: A Systematic Review and Meta-Analysis. Am. J. Gastroenterol..

[5] Moschen A.R., Tilg H., Raine T. (2019). IL-12, IL-23 and IL-17 in IBD: Immunobiology and therapeutic targeting. Nat. Rev. Gastroenterol. Hepatol..

[6] Iborra M., Beltrán B., Fernández-Clotet A., Gutiérrez A., Antolín B., Huguet J.M., De Francisco R., Merino O., Carpio D., García-López S. (2019). Real-world short-term effectiveness of ustekinumab in 305 patients with Crohn’s disease: Results from the ENEIDA registry. Aliment. Pharmacol. Ther..

[7] Biemans V.B.C., van der Meulen-de Jong A.E., van der Woude C.J., Löwenberg M., Dijkstra G., Oldenburg B., de Boer N.K.H., van der Marel S., Bodelier A.G.L., Jansen J.M. (2019). Ustekinumab for Crohn’s Disease: Results of the ICC Registry, a Nationwide Prospective Observational Cohort Study. J. Crohn’s Colitis.

[8] Battat R., Kopylov U., Bessissow T., Bitton A., Cohen A., Jain A., Martel M., Seidman E., Afif W. (2017). Association Between Ustekinumab Trough Concentrations and Clinical, Biomarker, and Endoscopic Outcomes in Patients With Crohn’s Disease. Clin. Gastroenterol. Hepatol..

[9] Ma C., Fedorak R.N., Kaplan G.G., Dieleman L.A., Devlin S.M., Stern N., Kroeker K.I., Seow C.H., Leung Y., Novak K.L. (2017). Clinical, endoscopic and radiographic outcomes with ustekinumab in medically-refractory Crohn’s disease: Real world experience from a multicentre cohort. Aliment. Pharmacol. Ther..

[10] Verstockt B., Dreesen E., Noman M., Outtier A., Van den Berghe N., Aerden I., Compernolle G., Van Assche G., Gils A., Vermeire S. (2019). Ustekinumab Exposure-outcome Analysis in Crohn’s Disease Only in Part Explains Limited Endoscopic Remission Rates. J. Crohn’s Colitis.

[11] Hanžel J., Zdovc J., Kurent T., Sever N., Javornik K., Tuta K., Koželj M., Smrekar N., Novak G., Štabuc B. (2021). Peak Concentrations of Ustekinumab After Intravenous Induction Therapy Identify Patients With Crohn’s Disease Likely to Achieve Endoscopic and Biochemical Remission. Clin. Gastroenterol. Hepatol..

[12] Ollech J.E., Normatov I., Peleg N., Wang J., Patel S.A., Rai V., Yi Y., Singer J., Dalal S.R., Sakuraba A. (2020). Effectiveness of Ustekinumab Dose Escalation in Patients With Crohn’s Disease. Clin. Gastroenterol. Hepatol..

[13] Kopylov U., Hanzel J., Liefferinckx C., De Marco D., Imperatore N., Plevris N., Baston-Rey I., Harris R.J., Truyens M., Domislovic V. (2020). Effectiveness of ustekinumab dose escalation in Crohn’s disease patients with insufficient response to standard-dose subcutaneous maintenance therapy. Aliment. Pharmacol. Ther..

[14] Fumery M., Peyrin-Biroulet L., Nancey S., Altwegg R., Gilletta C., Veyrard P., Bouguen G., Viennot S., Poullenot F., Filippi J. (2020). Effectiveness And Safety Of Ustekinumab Intensification At 90 Mg Every Four Weeks In Crohn’s Disease: A Multicenter Study. J. Crohn’s Colitis.

[15] Hanžel J., Koželj M., Špes Hlastec A., Kurent T., Sever N., Zdovc J., Smrekar N., Novak G., Štabuc B., Grabnar I. (2021). Ustekinumab concentrations shortly after escalation to monthly dosing may identify endoscopic remission in refractory Crohn’s disease. Eur. J. Gastroenterol. Hepatol..

[16] Van den Reek J.M.P.A., Coenen M.J.H., van de L’Isle Arias M., Zweegers J., Rodijk-Olthuis D., Schalkwijk J., Vermeulen S.H., Joosten I., van de Kerkhof P.C.M., Seyger M.M.B. (2017). Polymorphisms in CD84, IL12B and TNFAIP3 are associated with response to biologics in patients with psoriasis. Br. J. Dermatol..

[17] Galluzzo M., Boca A.N., Botti E., Potenza C., Malara G., Malagoli P., Vesa S., Chimenti S., Buzoianu A.D., Talamonti M. (2016). IL12B (p40) Gene Polymorphisms Contribute to Ustekinumab Response Prediction in Psoriasis. Dermatology.

[18] Rožman S., Novaković S., Grabnar I., Cerkovnik P., Novaković B.J. (2016). The impact of FcγRIIa and FcγRIIIa gene polymorphisms on responses to RCHOP chemotherapy in diffuse large B-cell lymphoma patients. Oncol. Lett..

[19] Janmahasatian S., Duffull S.B., Ash S., Ward L.C., Byrne N.M., Green B. (2005). Quantification of lean bodyweight. Clin. Pharmacokinet..

[20] Mager D.E., Jusko W.J. (2001). General pharmacokinetic model for drugs exhibiting target-mediated drug disposition. J. Pharmacokinet. Pharmacodyn..

[21] Gibiansky L., Gibiansky E. (2009). Target-mediated drug disposition model: Approximations, identifiability of model parameters and applications to the population pharmacokinetic–pharmacodynamic modeling of biologics. Expert Opin. Drug Metab. Toxicol..

[22] Ternant D., Monjanel H., Venel Y., Prunier-Aesch C., Arbion F., Colombat P., Paintaud G., Gyan E. (2019). Nonlinear pharmacokinetics of rituximab in non-Hodgkin lymphomas: A pilot study. Br. J. Clin. Pharmacol..

[23] Mager D.E., Krzyzanski W. (2005). Quasi-equilibrium pharmacokinetic model for drugs exhibiting target-mediated drug disposition. Pharm. Res..

[24] Nadeau R.R., Ostrowski C., Ni-Wu G., Liberato D.J. (1995). Pharmacokinetics and pharmacodynamics of recombinant human interleukin-12 in male rhesus monkeys. J. Pharmacol. Exp. Ther..

[25] Zhang T.T., Ma J., Durbin K.R., Montavon T., Lacy S.E., Jenkins G.J., Doktor S., Kalvass J.C. (2019). Determination of IL-23 Pharmacokinetics by Highly Sensitive Accelerator Mass Spectrometry and Subsequent Modeling to Project IL-23 Suppression in Psoriasis Patients Treated with Anti-IL-23 Antibodies. AAPS J..

[26] Zhang L., Beal S.L., Sheiner L.B. (2003). Simultaneous vs. Sequential Analysis for Population PK/PD Data I: Best-case Performance. Simulation.

[27] Felmlee M.A., Morris M.E., Mager D.E. (2012). Mechanism-Based Pharmacodynamic Modeling. Computational Toxicology.

[28] Bergstrand M., Karlsson M.O. (2009). Handling data below the limit of quantification in mixed effect models. AAPS J..

[29] Lin J.-F., Chen J.-M., Zuo J.-H., Yu A., Xiao Z.-J., Deng F.-H., Nie B., Jiang B. (2014). Meta-analysis: Fecal Calprotectin for Assessment of Inflammatory Bowel Disease Activity. Inflamm. Bowel Dis..

[30] Guidi L., Marzo M., Andrisani G., Felice C., Pugliese D., Mocci G., Nardone O., De Vitis I., Papa A., Rapaccini G. (2014). Faecal calprotectin assay after induction with anti-Tumour Necrosis Factor α agents in inflammatory bowel disease: Prediction of clinical response and mucosal healing at one year. Dig. Liver Dis..

[31] Adedokun O.J., Xu Z., Gasink C., Jacobstein D., Szapary P., Johanns J., Gao L.-L.L., Davis H.M., Hanauer S.B.S., Feagan B.G. (2018). Pharmacokinetics and Exposure Response Relationships of Ustekinumab in Patients With Crohn’s Disease. Gastroenterology.

[32] Hudson J., Herfarth H., Barnes E. (2020). Letter: Optimising response to ustekinumab therapy for patients with Crohn’s disease. Aliment. Pharmacol. Ther..

[33] Sedano R., Guizzetti L., McDonald C., Jairath V. (2020). Intravenous Ustekinumab Reinduction Is Effective in Prior Biologic Failure Crohn’s Disease Patients Already on Every-4-Week Dosing. Clin. Gastroenterol. Hepatol..

[34] Danese S., Vermeire S., D’Haens G., Panés J., Dignass A., Magro F., Nazar M., Le Bars M., Sloan S., Lahaye M. (2020). DOP13 Clinical and endoscopic response to ustekinumab in Crohn’s disease: Week 16 interim analysis of the STARDUST trial. J. Crohn’s Colitis.

[35] Zhu Y., Hu C., Lu M., Liao S., Marini J.C., Yohrling J., Yeilding N., Davis H.M., Zhou H. (2009). Population pharmacokinetic modeling of ustekinumab, a human monoclonal antibody targeting IL-12/23p40, in patients with moderate to severe plaque psoriasis. J. Clin. Pharmacol..

[36] Zhu Y.W., Mendelsohn A., Pendley C., Davis H.M., Zhou H. (2010). Population pharmacokinetics of ustekinumab in patients with active psoriatic arthritis. Int. J. Clin. Pharmacol. Ther..

[37] Xu Y., Hu C., Chen Y., Miao X., Adedokun O.J., Xu Z., Sharma A., Zhou H. (2020). Population Pharmacokinetics and Exposure-Response Modeling Analyses of Ustekinumab in Adults With Moderately to Severely Active Ulcerative Colitis. J. Clin. Pharmacol..

[38] Wang Z., Verstockt B., Sabino J., Vermeire S., Ferrante M., Declerck P., Dreesen E. (2021). Population pharmacokinetic-pharmacodynamic model-based exploration of alternative ustekinumab dosage regimens for patients with Crohn’s disease. Br. J. Clin. Pharmacol..

[39] Hu C., Adedokun O.J., Chen Y., Szapary P.O., Gasink C., Sharma A., Zhou H. (2017). Challenges in longitudinal exposure-response modeling of data from complex study designs: A case study of modeling CDAI score for ustekinumab in patients with Crohn’s disease. J. Pharmacokinet. Pharmacodyn..

[40] Berends S.E., van Steeg T.J., Ahsman M.J., Singh S., Brandse J.F., D’Haens G.R.A.M., Mathôt R.A.A. (2019). Tumor necrosis factor-mediated disposition of infliximab in ulcerative colitis patients. J. Pharmacokinet. Pharmacodyn..

[41] Dirks N.L., Meibohm B. (2010). Population Pharmacokinetics of Therapeutic Monoclonal Antibodies. Clin. Pharmacokinet..

[42] Ogawa K., Matsumoto T., Esaki M., Torisu T., Iida M. (2012). Profiles of circulating cytokines in patients with Crohn’s disease under maintenance therapy with infliximab. J. Crohn’s Colitis.

[43] Lucaciu L., Ilies M., Iuga C., Seicean A. (2018). P136 Serum IL-17 and IL-23 levels can distinguish between severe and non-severe inflammatory bowel disease. J. Crohn’s Colitis.

[44] Wong E.C.L., Marshall J.K., Reinisch W., Narula N. (2020). Body Mass Index Does Not Impact Clinical Efficacy of Ustekinumab in Crohn’s Disease: A Post Hoc Analysis of the IM-UNITI Trial. Inflamm. Bowel Dis..

[45] Sand K.M.K., Bern M., Nilsen J., Noordzij H.T., Sandlie I., Andersen J.T. (2015). Unraveling the Interaction between FcRn and Albumin: Opportunities for Design of Albumin-Based Therapeutics. Front. Immunol..

[46] Moroi R., Endo K., Kinouchi Y., Shiga H., Kakuta Y., Kuroha M., Kanazawa Y., Shimodaira Y., Horiuchi T., Takahashi S. (2013). FCGR3A-158 polymorphism influences the biological response to infliximab in Crohn’s disease through affecting the ADCC activity. Immunogenetics.

[47] Louis E., El Ghoul Z., Vermeire S., Dall’Ozzo S., Rutgeerts P., Paintaud G., Belaiche J., De Vos M., Van Gossum A., Colombel J.-F. (2004). Association between polymorphism in IgG Fc receptor IIIa coding gene and biological response to infliximab in Crohn’s disease. Aliment. Pharmacol. Ther..

[48] Castro-Dopico T., Clatworthy M.R. (2019). IgG and Fcγ receptors in intestinal immunity and inflammation. Front. Immunol..

[49] Dreesen E., Berends S., Laharie D., D’Haens G., Vermeire S., Gils A., Mathôt R. (2020). Modelling of the relationship between infliximab exposure, faecal calprotectin and endoscopic remission in patients with Crohn’s disease. Br. J. Clin. Pharmacol..

[50] Feagan B.G., Sandborn W.J., Gasink C., Jacobstein D., Lang Y., Friedman J.R., Blank M.A., Johanns J., Gao L.L., Miao Y. (2016). Ustekinumab as induction and maintenance therapy for Crohn’s disease. N. Engl. J. Med..

[51] Hanauer S.B., Sandborn W.J., Feagan B.G., Gasink C., Jacobstein D., Zou B., Johanns J., Adedokun O.J., Sands B.E., Rutgeerts P. (2020). IM-UNITI: Three-year efficacy, safety, and immunogenicity of ustekinumab treatment of Crohn’s disease. J. Crohn’s Colitis.

[52] Sands B.E., Sandborn W.J., Panaccione R., O’Brien C.D., Zhang H., Johanns J., Adedokun O.J., Li K., Peyrin-Biroulet L., Van Assche G. (2019). Ustekinumab as Induction and Maintenance Therapy for Ulcerative Colitis. N. Engl. J. Med..

[53] Labaere D., Smismans A., Van Olmen A., Christiaens P., D’Haens G., Moons V., Cuyle P.-J., Frans J., Bossuyt P. (2014). Comparison of six different calprotectin assays for the assessment of inflammatory bowel disease. United Eur. Gastroenterol. J..

