# Supplementary material for: Ustekinumab Dosing Individualization in Crohn’s Disease Guided by a Population Pharmacokinetic–Pharmacodynamic Model
Source: Pharmaceutics. 2021 Sep 30;13(10):1587. doi: 10.3390/pharmaceutics13101587 (PMC8538292; doi:10.3390/pharmaceutics13101587)
Supplement: Supplementary file 1 [file pharmaceutics-13-01587-s001.zip › pharmaceutics-1390381-supplementary.pdf]

pharmaceutics

Supplementary Materials: Ustekinumab Dosing Individualization in Crohn's Disease Guided by a Population Pharmacokinetic-Pharmacodynamic Model

Jurij Aguiar Zdovc, Jurij Hanžel, Tina Kurent, Nejc Sever, Matic Koželj, Nataša Smrekar, Gregor Novak, Borut Štabuc, Erwin Dreesen, Debby Thomas, Tomaž Vovk, Barbara Ostanek, David Drobne and Iztok Grabnar

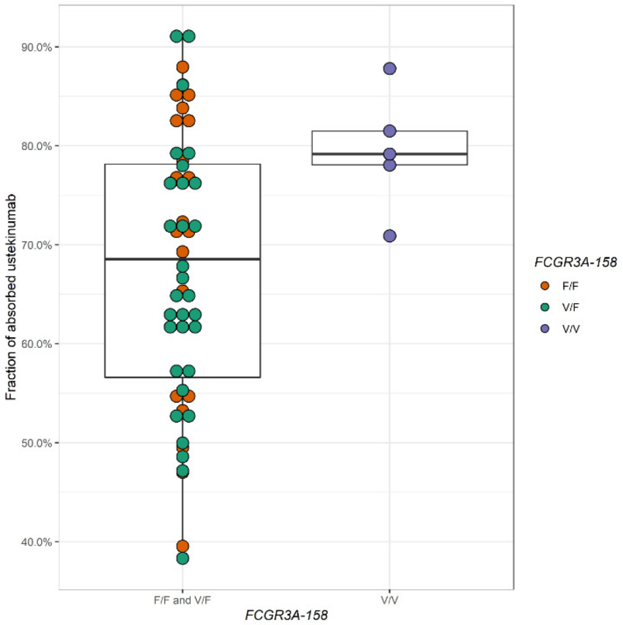

Figure S1. Individual estimates (points) of fraction of absorbed ustekinumab after subcutaneous administration calculated by the base pharmacokinetic model in patients with *FCGR3A* rs396991 polymorphism (*FCGR3A*-158) phenylalanine/phenylalanine (F/F, orange) or valine/phenylalanine (V/F, green) variant, compared to patients with valine/valine (V/V, purple) variant. Median (line across boxes), 1st and 3rd quartile (lower and upper hinges), minimum and maximum non-outlying (< 1.5 times interquartile range) values (whiskers).

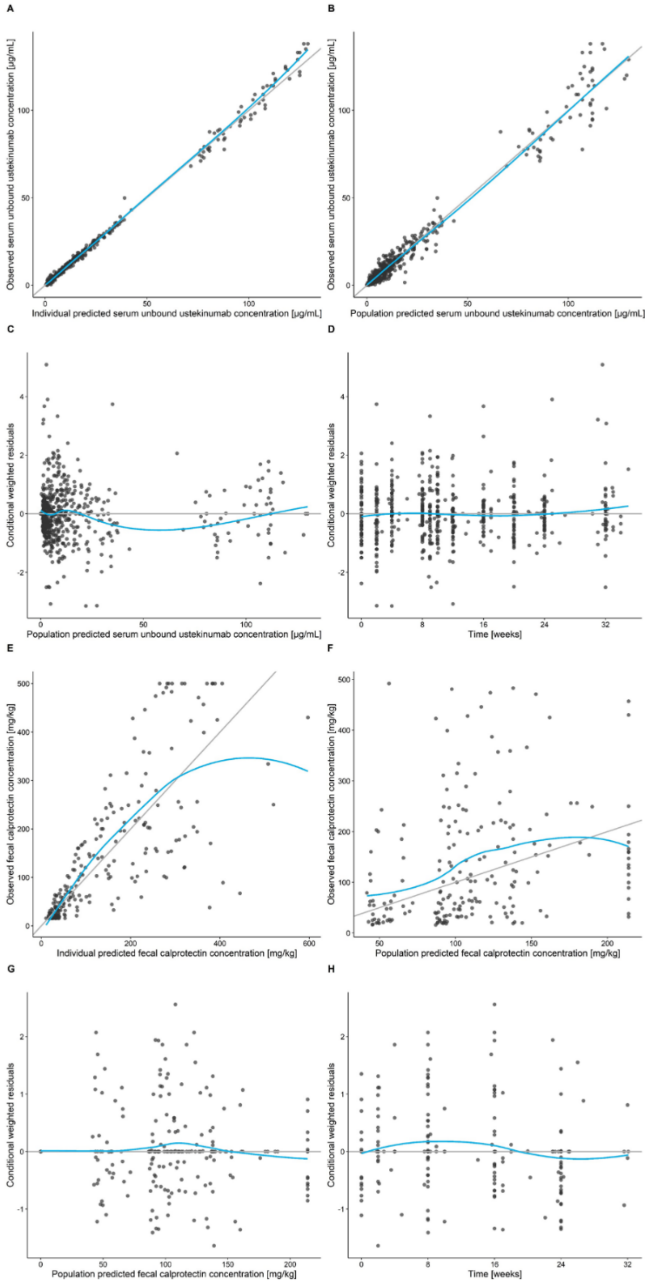

Figure S2. Diagnostic plots of the final pharmacokinetic (A-D) and pharmacodynamic (E-H) model: (A) observed vs individual model-predicted unbound ustekinumab concentrations (points), LOESS fit (blue) and identity line (grey); (B) observed vs population model-predicted unbound ustekinumab concentrations (points), LOESS fit (blue) and identity line (grey). (C) Conditional weighted residuals vs population predicted unbound ustekinumab concentrations (points) and LOESS fit line (blue); (D) conditional weighted residuals vs time since first dose (points) and LOESS fit line (blue); (E) observed vs individual model-predicted fecal calprotectin concentrations (points), LOESS fit (blue) and identity line (grey); (F) observed vs population model-predicted fecal calprotectin concentrations (points), LOESS fit (blue) and identity line (grey). (G) Conditional weighted residuals vs population predicted fecal calprotectin concentrations (points) and LOESS fit line (blue); (H) conditional weighted residuals vs time since first dose (points) and LOESS fit line (blue).

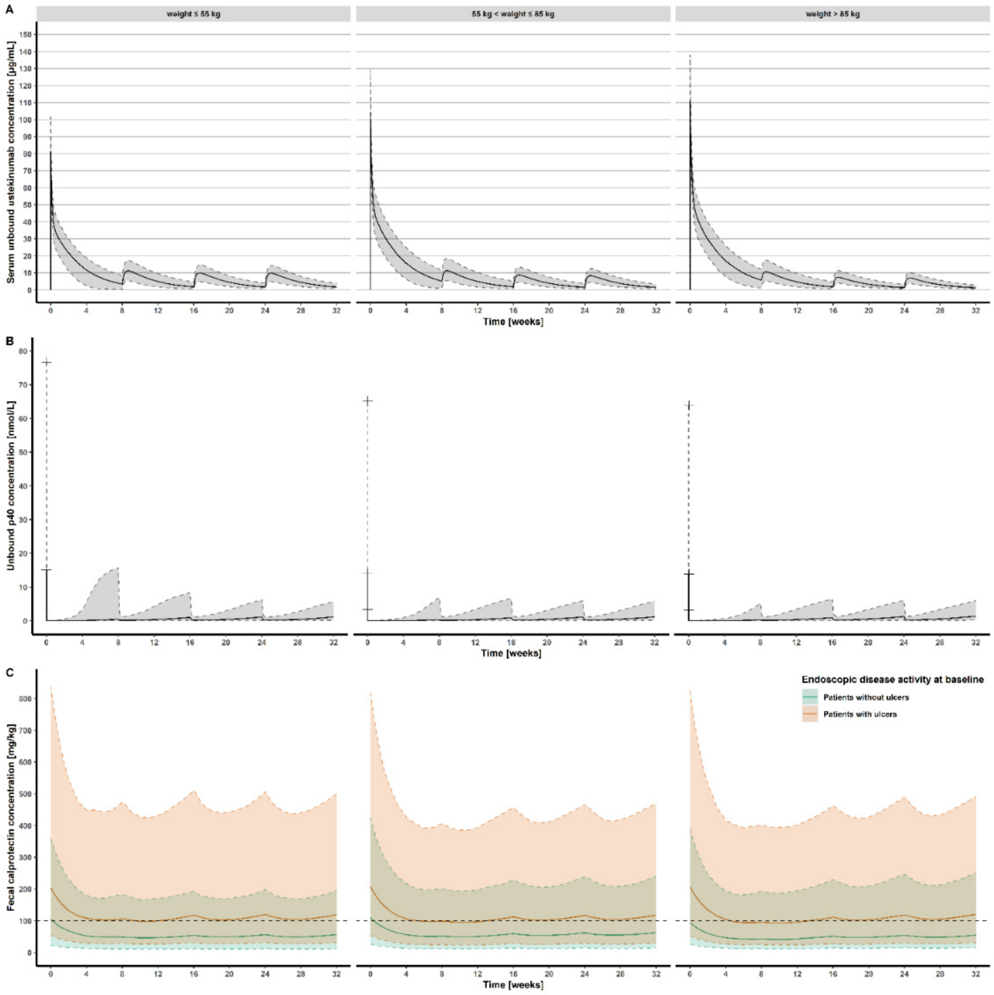

Figure S3. A population simulation using the final pharmacokinetic–pharmacodynamic model and representative virtual patient population (n = 10000). A) Median serum unbound ustekinumab concentration (bold line), and 95% prediction interval (grey shaded area) over time in patients with body weight: ≤ 55 kg (left); > 55 kg and ≤ 85 kg (middle); and > 85 kg (right); B) Median serum unbound p40 concentration (bold line) and 95% prediction interval (grey shaded area) over time in patients with body weight: ≤ 55 kg (left); > 55 kg and ≤ 85 kg (middle); and > 85 kg (right); C) Median (bold lines) fecal calprotectin concentration and 95% prediction interval (shaded area) in patients with ulcers (orange) and without ulcers (green) at baseline, with body weight: ≤ 55 kg (left); > 55 kg and ≤ 85 kg (middle); and > 85 kg (right).

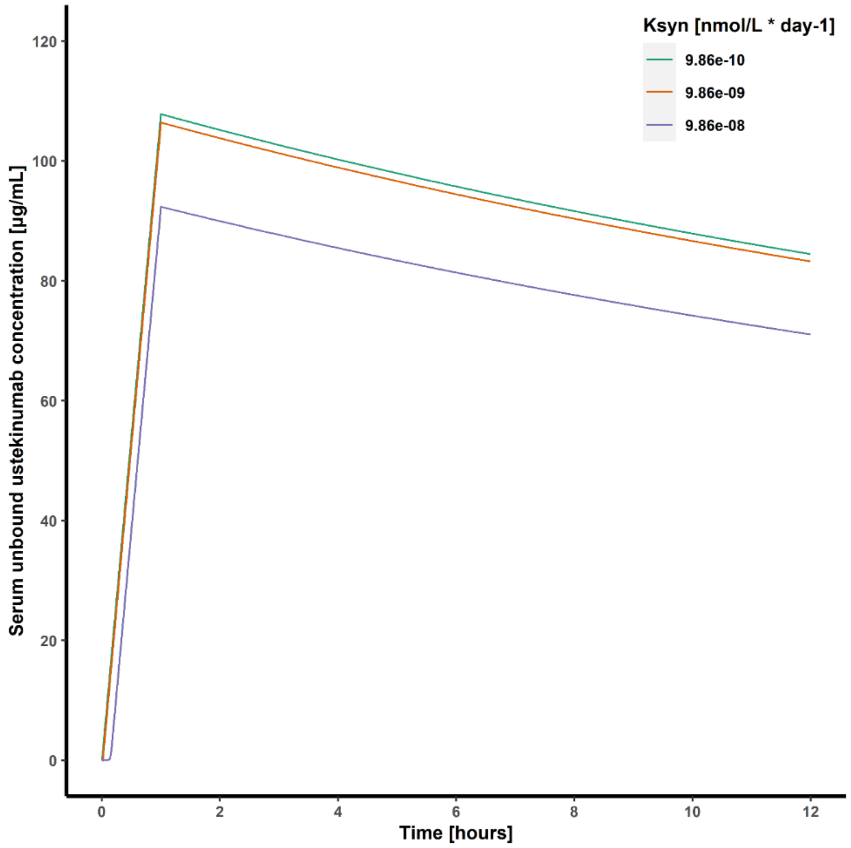

Figure S4. Serum unbound ustekinumab concentration time-profile in a patient with high (purple), typical (orange), or low (green) target synthesis rate constant (K_{syn}) and typical values of other pharmacokinetic parameters.

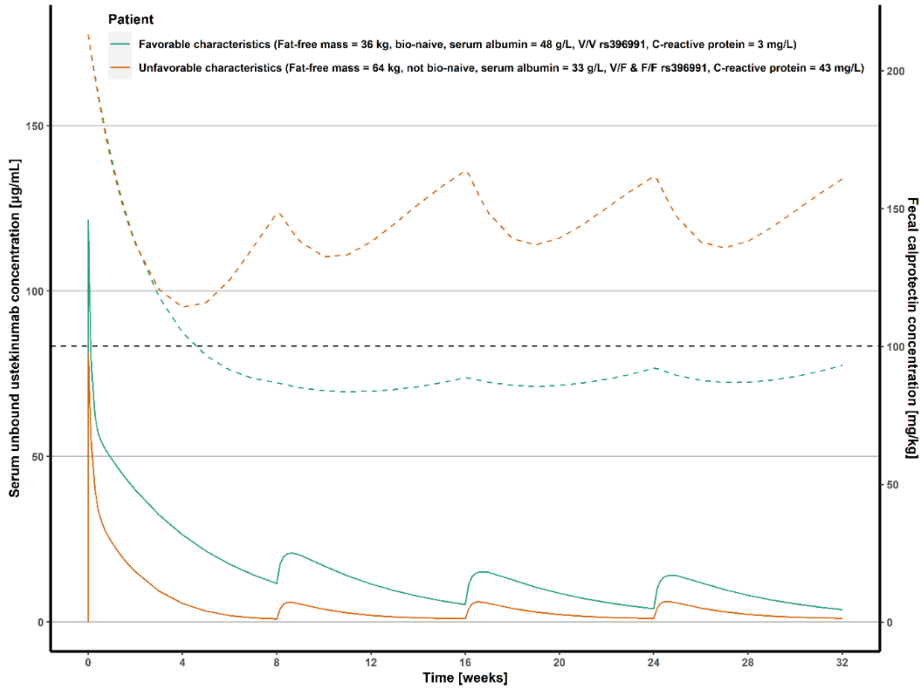

Figure S5. Simulated ustekinumab (full) and fecal calprotectin (dashed) concentration time-profile of a typical patient with covariates associated with higher ustekinumab exposure and lower target synthesis (fat-free mass = 36 kg, bio-naïve, serum albumin = 48 g/L, V/V genotype of *FCGR3A*-158 and serum C-reactive protein = 3 mg/L; green) and a typical patient with covariates associated with lower ustekinumab exposure and higher target synthesis (fat-free mass = 64 kg, non-bio-naïve, serum albumin = 33 g/L, V/F or F/F genotype of rs396991 and serum C-reactive protein = 43 mg/L; orange). Both patients had active disease at baseline and received a baseline intravenous induction dose of 390 mg, followed by fixed subcutaneous maintenance doses of 90 mg, administered every eight weeks. The extremes of covariate values observed in patients with body weight > 55 kg and ≤ 85 kg in the study cohort were assumed.

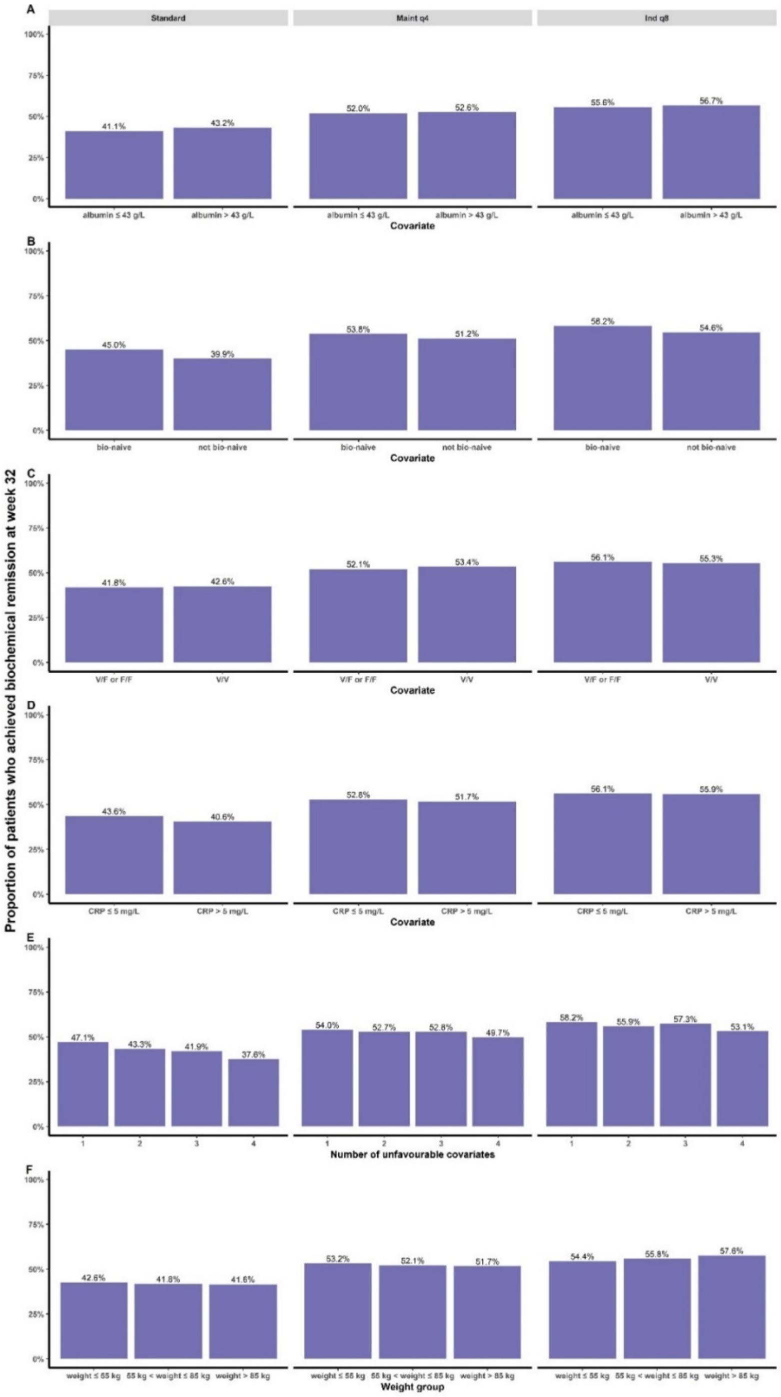

Figure S6. Proportion of patients who achieved biochemical remission at week 32, stratified by each covariate (A-D), by number of unfavorable covariates (E) and by weight groups according to the induction dose (F) for standard treatment (Standard), weight-based induction dose at baseline followed by fixed 90 mg maintenance dosing every four weeks (Maint q4), or weight-based induction dosing every eight weeks (Ind q8). Unfavorable covariates were defined as albumin ≤ 43 g/L (A), not bio-naïve (B), V/F or F/F variant of *FCGR3A*-158 polymorphism (C) and C-reactive protein > 5 mg/L (D).

Table S1. Comparison of the tested base pharmacokinetic models.

Copyright: © 2021 by the authors. Licensee MDPI, Basel, Switzerland. This article is an open access article distributed under the terms and conditions of the Creative Commons Attribution (CC BY) license (https://creativecommons.org/licenses/by/4.0/).

**Model**	**Estimated parameters**	**Number of disposition compartments**	**AIC**
Linear models			
One-compartment	K_a, CL, V_c, F, IIV on CL, IIV on V_c, IIV on F	1	4005.3
Two-compartment	K_a, CL, V_c, Q, V_p, F, IIV on CL, IIV on V_c, IIV on F	2	3621.4
TMDD models			
Exponential CL decay over time	K_a, CL_1, V_c, Q, V_p, K_{des}, CL_2, F, IIV on CL_1, IIV on V_c, IIV on F	2	3600.6
Michaelis-Menten type	K_a, CL, V_c, Q, V_p, K_m, V_m, F, IIV on CL, IIV on V_c, IIV on F	2	3611.2
Irreversible binding with constant target (no target turnover)	K_a, CL, V_c, Q, V_p, R_0, K_{int}, F, IIV on CL, IIV on V_c, IIV on F	3	3625.5
Irreversible binding with target turnover	K_a, CL, V_c, Q, V_p, K_{syn}, K_{deg}, K_{int}, F, IIV on CL, IIV on V_c, IIV on F	3	3620.6
Quasi-equilibrium	K_a, CL, V_c, Q, V_p, K_{syn}, K_{deg}, K_{int}, K_d, F, IIV on CL, IIV on V_c, IIV on F	3	3581.6
Quasi-equilibrium including a peripheral compartment for target distribution	K_a, CL, V_c, Q, V_p, K_{syn}, K_{deg}, K_{int}, K_d, F, $V_{c\text{-}target}$, Q_{target}, $V_{p\text{-}target}$, IIV on CL, IIV on V_c, IIV on F	4	3574.8
Quasi-equilibrium with a peripheral compartment for target distribution and additional IIV	K_a, CL, V_c, Q, V_p, K_{syn}, K_{deg}, K_{int}, K_d, F, $V_{c\text{-}target}$, Q_{target}, $V_{p\text{-}target}$, IIV on CL, IIV on V_c, IIV on V_p, IIV on F, IIV on K_{syn}	4	3523.0

AIC – Akaike information criterion; Ka – ustekinumab absorption rate constant after subcutaneous administration; CL – clearance of ustekinumab, Vc – volume of distribution in central compartment; F – fraction of absorbed ustekinumab after subcutaneous administration; IIV – interindividual variability; TMDD – target-mediated drug disposition; Q – intercompartmental clearance, Vp – volume of distribution in peripheral compartment; CL1 – linear clearance; Kdes – total clearance decrease rate constant; CL2 – initial target-mediated nonlinear clearance; Km – Michaelis constant; Vm – maximum elimination rate; R0 – initial target concentration; Kint – elimination rate constant due to binding; Ksyn – rate constant of target synthesis; Kdeg –rate constant of target degradation; Kd – equilibrium constant.

Supplementary information, S1: NONMEM control stream for the population PK and PD model.

Population PK model.

```
$PROBLEM      PopPK analysis of ustekinumab
$INPUT        ID DAT1=DROP TIME RATE AMT CMT DV BQL EVID MDV HT WT FFM SEX AGE Albumin BASECRP SMOK
DISDUR NAIVE AZA rs3212227 rs3213094 rs6887695 rs1801274 rs396991
$DATA Ustekinumab_Data_1.csv IGNORE=#
$SUBROUTINE ADVAN13 TOL9
$MODEL        COMP=(DEPOT)     ; 1
              COMP=(CENTR)     ; 2
              COMP=(PERIPH)    ; 3
              COMP=(TRGTC)     ; 4
              COMP=(TRGTP)     ; 5
              COMP=(AUCF)      ; 6
$PK

IF(NAIVE.EQ.0) CL1NAIVE = 1   ; Most common
IF(NAIVE.EQ.1) CL1NAIVE = ( 1 + THETA(16))
CL1FFM = ((FFM/45)**THETA(17))
IF(Albumin.EQ.-99) THEN CL1Albumin = 1
ELSE CL1Albumin = ( 1 + THETA(18)*(Albumin - 43))
ENDIF

V2FFM = ((FFM/45)**THETA(19))
V3FFM = ((FFM/45)**THETA(20))

IF(rs396991.LT.3) F1rs396991 = THETA(6)   ; Most common
IF(rs396991.EQ.3) F1rs396991 = THETA(21)

IF(BASECRP.EQ.-99) THEN KsynCRP = 1
ELSE KsynCRP = ( 1 + THETA(22)*(BASECRP - 3))
ENDIF

CL1COV=CL1NAIVE*CL1FFM*CL1Albumin
KsynCOV = KsynCRP

KA=THETA(1)
TVCL1= CL1COV*THETA(2)
CL1=TVCL1*EXP(ETA(2))
TVV2= V2FFM*THETA(3)
```

```
V2=TVV2*EXP(ETA(3))
TVV3= V3FFM*THETA(4)
V3=TVV3*EXP(ETA(4))
Q=THETA(5)
F1=EXP(LOG(F1rs396991/(1-F1rs396991))+ETA(5))/(EXP(LOG(F1rs396991/(1-F1rs396991))+ETA(5))+1)
TVKsyn= KsynCOV*THETA(7)
Ksyn= TVKsyn*EXP(ETA(6))
Kdeg= THETA(8)
Kd=THETA(9)
Kint=THETA(10)
QT=THETA(11)
VCT=THETA(12)
VPT=THETA(13)

K12T = QT/VCT
K21T = QT/VPT
VPT = K12T*VCT/K21T

A_0(4)= Ksyn/Kdeg
A_0(5) = Ksyn*VPT/Kdeg

S2=V2

$DES

Ct = A(2)/V2                          ; total ustekinumab concentration
D = Ct - A(4) - Kd                    ; abbreviation of the next line
C = 0.5*(D+SQRT(D**2+4*Kd*Ct))        ; unbound ustekinumab concentration
P40 = A(4)/1                          ; total target concentration
P40free = P40*Kd/(Kd+C)               ; unbound target concentration

DADT(1)= -KA*A(1)
DADT(2)= KA*A(1) - (Q/V2)*C*V2 + (Q/V3)*A(3) - (CL1/V2)*C*V2 - Kint*A(4)*C*V2 /(Kd+C)
DADT(3)= (Q/V2)*C*V2 - (Q/V3)*A(3)
DADT(4)= Ksyn - Kdeg*A(4) - (Kint-Kdeg)*C*A(4)/(Kd+C) - K12T*P40free + K21T*A(5)/VCT
DADT(5) =   K12T*P40free*VCT - K21T*A(5)
DADT(6)= C

AUCF = A(6)

$ERROR
Ctot = A(2)/V2                        ; total ustekinumab concentration
```

```
Rtot =A(4)                          ; total target concentration
DD = Ctot - A(4) - Kd               ; abbreviation of the next line
Cfree = 0.5*(DD+SQRT(DD**2+4*Kd*Ctot))      ; unbound ustekinumab concentration [nmol/L]
Cfreemg = 0.149*Cfree                       ; unbound ustekinumab concentration [mg/L]
RC = Rtot*Cfree/(Kd+Cfree)                  ; complex concentration
R = Rtot*Kd/(Kd+Cfree)                      ; unbound target concentration
IPRED = Cfree                               ; fitting the unbound ustekinumab concentration
W = SQRT(THETA(14)**2+(THETA(15)*IPRED)**2) ; a combination residual error

LLOQ=2.349 ; in nmol/L
DUM1=(LLOQ-IPRED)/W
CUMD1=PHI(DUM1)

IF(BQL.EQ.0) THEN
F_FLAG = 0
Y = IPRED+EPS(1)*W

ENDIF
IRES   = IPRED-DV
IWRES = IRES/W

IF(BQL.EQ.1) THEN
F_FLAG = 1
Y=CUMD1
ENDIF

$THETA
(0, 0.0159) ; KA (h-1) ; time in hours
(0, 0.0115) ; CL (L/h)
(0, 3.57) ; V2   (L)
(0, 3.30) ; V3   (L)
(0, 0.0787) ; Q (L/h)
(0, 0.710) ; F1 (.)
(0, 4.11E-10) ; Ksyn (nmol/(L*h)))
(0, 3.86E-11) ; Kdeg (h-1)
(0, 0.168) ; Kd (nmol/L)
(0, 1.18E-07) ; Kint (h-1)
(0, 0.0205) ; QT (L/h)
(0, 2.44) ; VCT   (L)
(0, 11.0) ; VPT   (L)
(0, 4.55) ; Wa (nmol/L)
(0, 0.0777) ; Wp (.)
```

```
(-1, -0.227,5) ; CL1NAIVE1
(0, 0.598,100000) ; CL1FFM
(-0.143, -0.0165,0.056) ; CL1Albumin1
(0, 0.590,100000) ; V2FFM
(0, 0.586,100000) ; V3FFM
(0, 0.888,20) ; F1rs396991
(0, 0.0846,10) ; KsynCRP

$OMEGA 0 FIX   ;        IIV_KA
$OMEGA 0.0320 ;        IIV_CL
$OMEGA 0.00955 ;        IIV_V2
$OMEGA 0.0565 ;        IIV_V3
$OMEGA 0.703 ;        IIV_F1
$OMEGA 0.685   ;     IIV_Ksyn
$SIGMA 1 FIX   ; EPS(1)

$ESTIMATION
SIGDIGITS=3 MAXEVALS=9999 REPEAT METHOD=1 LAPLACIAN INTERACTION NUMERICAL SLOW
POSTHOC
PRINT=5
NOABORT

$COVARIANCE PRINT=E
```

Population PK-PD model

```
$PROBLEM      PopPK-PD analysis of ustekinumab
$INPUT          ID DAT1=DROP TIME RATE AMT DV EVID MDV BQL UQL SESCD BASEULCERS IKA ICL1 IV2 IV3 IQ IF1
IKINT IKSYN IKDEG IKD IQT IVCT IVPT
$DATA          Ustekinumab_Data_2.csv IGNORE=#
$SUBROUTINE ADVAN13 TOL9
$MODEL          COMP=(DEPOT)
                COMP=(CENTR)
                COMP=(PERIPH)
                COMP=(EFF)
                COMP=(TRGTC)
                COMP=(TRGTP)
                COMP=(AUCF)
$PK
KA = IKA
```

```
CL1 = ICL1
V2 = IV2
V3 = IV3
Q = IQ
F1 = IF1
Kint=IKINT
Ksyn=IKSYN
Kdeg=IKDEG
Kd=IKD
QT = IQT
VCT = IVCT
VPT = IVPT
K12T = QT/VCT
K21T = QT/VPT

S2 = V2

IF(BASEULCERS.EQ.0) C0Ulcers = THETA(4)    ; Most common
IF(BASEULCERS.EQ.1) C0Ulcers = THETA(5)

Kout = THETA(1)
EMAX=THETA(2)
C50=THETA(3)

TVC0=C0Ulcers
C0=TVC0*EXP(ETA(1))

P0 = Ksyn/Kdeg
EFF0 = EMAX*P0/(C50+P0)
A_0(4) = C0
A_0(5) = P0
A_0(6) = P0*VPT

KIN = C0*Kout/(1+EFF0)

$DES
Ct = A(2)/V2
D = Ct - A(5) - Kd
C = 0.5*(D+SQRT(D**2+4*Kd*Ct))
```

```
P40 = A(5)/1
P40free = P40*Kd/(Kd+C) ; free target concentration

DADT(1)= -KA*A(1)
DADT(2)= KA*A(1) - (Q/V2)*C*V2 + (Q/V3)*A(3) - (CL1/V2)*C*V2 - Kint*A(5)*C*V2 /(Kd+C)
DADT(3)= (Q/V2)*C*V2 - (Q/V3)*A(3)
DADT(5)= Ksyn - Kdeg*A(5) - (Kint-Kdeg)*C*A(5)/(Kd+C) - K12T*P40free + K21T*A(6)/VCT
DADT(6) =   K12T*P40free*VCT - K21T*A(6)
DADT(7)= C
AUCF = A(7)

EFF = EMAX*P40free/(C50+P40free)

DADT(4)   =   KIN*(1+EFF) - Kout*A(4)

$ERROR

IPRED = A(4)
WE      = SQRT(THETA(6)**2+(THETA(7)*IPRED)**2)

ULOQ=500
LLOQ=15.6

DUM1=(IPRED-ULOQ)/WE
CUMD1=PHI(DUM1)

DUM2=(LLOQ-IPRED)/WE
CUMD2=PHI(DUM2)

IF(UQL.EQ.0.AND.BQL.EQ.0) THEN
F_FLAG = 0
Y       = IPRED+EPS(1)*WE
ENDIF

IRES   = IPRED-DV
IWRES = IRES/WE

IF(UQL.EQ.1) THEN
```

```
F_FLAG = 1
Y=CUMD1
ENDIF

IF(BQL.EQ.1) THEN
F_FLAG = 1
Y=CUMD2
ENDIF

$THETA
 (0,0.00242) ; Kout (h-1)
 (0,2.19) ; EMAX (.)
 (0,2.46) ; C50 (nmol/L)
 (0,102) ; C0 (mg/kg)
 (0,213) ; C0Ulcers (mg/kg)
  0.00001 FIX ; WEA
 (0,0.573) ; WEP (.)

$OMEGA   0.683 ; IIV_C0
$SIGMA   1   FIX
$ESTIMATION MAXEVALS=9999 REPEAT METHOD=1 LAPLACIAN INTERACTION NUMERICAL SLOW NSIG=3 SIGL=9
PRINT=3 MCETA=100 POSTHOC NOABORT
$COVARIANCE SLOW
```
